# Predictors of Appropriate ICD Therapy in Patients with Arrhythmogenic Right Ventricular Cardiomyopathy: Long Term Experience of a Tertiary Care Center

**DOI:** 10.1371/journal.pone.0039584

**Published:** 2012-09-27

**Authors:** Pia K. Schuler, Laurent M. Haegeli, Ardan M. Saguner, Thomas Wolber, Felix C. Tanner, Rolf Jenni, Natascia Corti, Thomas F. Lüscher, Corinna Brunckhorst, Firat Duru

**Affiliations:** 1 Clinic for Cardiology, Cardiovascular Center, University Hospital of Zurich, Zurich, Switzerland; 2 Adult Congenital and Valvular Heart Disease Center, Department of Cardiology and Angiology, University Hospital Muenster, Muenster, Germany; 3 Center for Integrative Human Physiology, University of Zurich, Zurich, Switzerland; 4 Division of Clinical Pharmacology and Toxicology, University Hospital of Zurich, Zurich, Switzerland; Battista Hospital, Italy

## Abstract

**Introduction:**

Arrhythmogenic right ventricular cardiomyopathy (ARVC) is a rare genetically transmitted disease prone to ventricular arrhythmias. We therefore investigated the clinical, echocardiographical and electrophysiological predictors of appropriate implantable cardioverter defibrillator (ICD) therapy in patients with ARVC.

**Methods:**

A retrospective analysis was performed in 26 patients (median age of 40 years at diagnosis, 21 males and 5 females) with ARVC who underwent ICD implantation.

**Results:**

Over a median (range) follow-up period of 10 (2.7, 37) years, appropriate ICD therapy for ventricular arrhythmias was documented in 12 (46%) out of 26 patients. In all patients with appropriate ICD therapy the ICD was originally inserted for secondary prevention. Median time from ICD implantation to ICD therapy was 9 months (range 3.6, 54 months). History of heart failure was a significant predictor of appropriate ICD therapy (p = 0.033). Left ventricular disease involvement (p = 0.059) and age at implantation (p = 0.063) were borderline significant predictors. Patients with syncope at time of diagnosis were significantly less likely to receive ICD therapy (p = 0.02). Invasive electrophysiological testing was not significantly associated with appropriate ICD therapy.

**Conclusion:**

In our cohort of patients with ARVC, history of heart failure was a significant predictor of appropriate ICD therapy, whereas left ventricular involvement and age at time of ICD implantation were of borderline significance. These predictors should be tested in larger prospective cohorts to optimize ICD therapy in this rare cardiomyopathy.

## Introduction

Arrhythmogenic right ventricular cardiomyopathy (ARVC) is an inheritable cardiomyopathy with typically autosomal dominant inheritance pathologically characterized by progressive fibrofatty replacement of the right ventricular myocardium. This type of cardiomyopathy is a major cause of sudden cardiac death in young adults and athletes [1], [2], [3], [4], [5], [6], [7], [8], [9]. The clinical manifestation of ARVC varies considerably [10]. Cardiac electrical instability may be associated with ventricular arrhythmia leading to sudden cardiac death (SCD) [5], [6], [7]. Progressive structural involvement of the right, or both right and left ventricular myocardium leads to heart failure [11], [12].

Implantable cardioverter defibrillators (ICD) are increasingly utilized in patients with ARVC who have survived SCD or have developed ventricular tachyarrhythmias associated with syncope [13]. However, there are little data available to help identifying ARVC patients in whom the prophylactic implantation of an ICD is truly warranted [14], [15]. It has been previously demonstrated that ARVC patients who have extensive right ventricular dilatation and/or left ventricular involvement confirmed by echocardiography or cardiac magnetic resonance imaging, and those who had hemodynamic significant ventricular tachycardia, as well as patients with affected relatives having survived a SCD are at increased risk and may benefit from ICD therapy [16], [17], [18], [19], [20]. This study aimed to determine clinical, echocardiographical and electrophysiological predictors of appropriate ICD therapy in our cohort of ARVC patients.

## Methods

### Study population

Patients who were diagnosed with ARVC at the University Hospital of Zurich (Switzerland) based on the Task Force Criteria and in whom an ICD was implanted were enrolled in the study [21], [22], [23]. According to the local legislation, approval from the local ethical committee as well as patient informed consent were not required for this retrospective data analysis. Patients with other concomitant heart diseases, such as coronary heart disease or dilated cardiomyopathy, were excluded. Findings of surface electrocardiogram, transthoracic echocardiogram and invasive electrophysiological study were analyzed and compared in two outcome groups: patients who received appropriate ICD therapy and patients who did not require appropriate ICD therapy during follow-up.

### Clinical characteristics

The presence and nature of symptoms were collected at the time of ARVC diagnosis. In addition, patient charts were meticulously screened for past history of heart failure, which was defined by the presence of the following clinical features: symptoms of heart failure described typically by shortness of breath according to the New York Heart Association (NYHA) functional classification and/or fatigue; physical signs of fluid retention, such as pulmonary congestion or ankle swelling; and objective evidence of a structural or functional cardiac abnormality as cardiomegaly, third heart sound, abnormality on the echocardiogram, and elevated natriuretic peptide concentration.

### Transthoracic echocardiography

Transthoracic echocardiograms were performed by an experienced operator in a standard fashion by two dimensional and Doppler echocardiography and M-mode measurements [24], [25], [26]. Left ventricular ejection fraction was calculated with the biplane area-length method [27]. Right ventricular morphology was evaluated from multiple views. All measurements were obtained from the apical four chamber view: right ventricular end-diastolic short axis diameter in the middle third of the right ventricle; and right ventricular end-diastolic (area D) and end-systolic area (area S). The fractional area change (normal >25%) of the right ventricle was defined as the ratio between the difference of the end-diastolic and end-systolic right ventricular areas and the end-diastolic area (that is, fractional area change  =  [area D – area S]/area D). The measurements were corrected for the body surface area. Left ventricular involvement of the disease was considered to be present either in the presence of reduced ejection fraction (<50%), if other causes were excluded, or in the presence of regional left ventricular dysplastic areas (aneurysm and localized wall thinning) [28].

### ICD therapy

**Figure 1:**
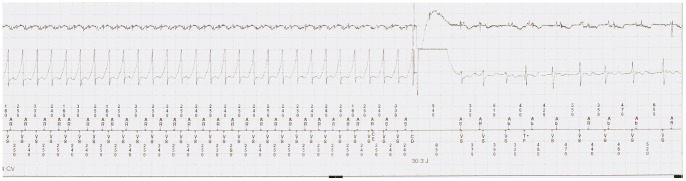
Intracardiac electrogram recording of a 49-year-old woman with ARVC and an implanted dual-chamber ICD. A sustained monomorphic ventricular tachycardia with a rate of 250 beats/min is appropriately detected by he device and terminated by an appropriate shock (30.3 Joule).

A cardioversion/defibrillation therapy or anti-tachycardia pacing for a ventricular arrhythmia faster than the programmed detection rate and accurately detected by the ICD, and confirmed by an experienced electrophysiologist at the University Hospital Zurich, was categorized as an appropriate ICD therapy (figure 1).

### Invasive electrophysiological study

A standardized ventricular stimulation protocol was performed in 16 patients out of 26 patients (62%) in the absence of antiarrhythmic drugs. The protocol consists in the delivery of up to three extrastimuli at three different basic drive train cycle lengths and ventricular burst pacing at two right ventricular sites (right ventricular apex and right ventricular outflow tract). Sustained ventricular tachycardia (VT) was defined as tachycardia of ventricular origin with a duration of more than 30 seconds or ventricular tachycardia leading to hemodynamic compromise.

### Statistical analysis

Continuous variables were summarized as median (range or interquartile range, as stated) since these mostly had skew distributions. Logistic models were used to estimate the odds associated with ICD therapy. Odds ratios are presented with 95% confidence intervals. Where there were zero events in some cells, the odds ratios and confidence intervals were estimated by adding 0.5 to each cell and Fishers exact testing used to assess significance. For skew data, the significance levels were also estimated using Mann-Whitney U-test between the two ICD groups.

## Results

Clinical data were collected in a total of 26 patients with ARVC (median age of 40 years at time of diagnosis, 21 males and 5 females) in whom an ICD was implanted. Table 1 summarizes the demographic data of the patient cohort. Patients were followed for a median duration of 10.7 years (patients with shock 14±10, patients with no shock 9.8±9.7 years). All patients received an ICD for secondary prevention of SCD due to documented ventricular tachycardia or fibrillation and/or an aborted SCD.

**Table 1:** Demographic and clinical parameters of total patient population.

Patients characteristics	Number or Median (Inter-quartile range)
(n = 26, unless stated otherwise)	
Men	21
Family history of ARVC	4
Age at diagnosis (years)	39.5 (35.75, 48.50)
Symptoms at time of ARVC diagnosis	
- aborted SCD	4
- syncope	12 (n = 24)
- heart failure	1 (n = 25)
- VT	19 (n = 25)
History of heart failure	4
Arrhythmia	
- atrial fibrillation or flutter	3
- VT	19 (n = 25)
- others (WPW, AVNRT)	3
Drug therapy	
- betablocker	14
- Amiodarone	5
- ACEI or ARB	5
ICD implantation	
- primary prevention	1
- secondary prevention	25
- age at ICD implantation	43.5 (36.75, 56.25)
- time period from ARVC diagnosis to ICD	1.5 months (2.5 days, 6.5 years)
- Implantation	
ICD therapy	
- appropriate therapy	12
- time from ICD implantation to first appropriate ICD therapy	9 months (3.6, 54 months)

(**VT**: ventricular tachycardia. **WPW**: Wolff-Parkinson-White-Syndrome; **AVNRT**: atrioventricular nodal re-entrant tachycardia; **ACEI**: angiotensin-converting enzyme inhibitor; **ARB**: angiotensin receptor blocker).

### Appropriate ICD therapy

12 out of the 26 patients (46%) received appropriate ICD therapy. The majority of the patients (n = 9) experienced the first appropriate therapy within the first 2 years after device implantation. Two patients received the first shock after 4 years, and one patient 8 years after device implantation. The median time period between the implantation of the ICD and the first appropriate ICD therapy was 9 months (range 2 months; 8 years). Multiple ICD therapies occurred in one patient due to pacing-induced ventricular proarrhythmia, which could be successfully prevented by device reprogramming.

### Clinical predictors for appropriate ICD therapy

The mean age at time of ARVC diagnosis was similar for patients with appropriate ICD therapy and patients without appropriate ICD therapy. Table 2 summarizes the clinical data in both groups. No significant difference in the presenting symptoms at time of ARVC diagnosis were observed between both groups except for syncope where there were 9 patients without ICD therapy versus only 3 in the patient group with appropriate ICD therapy (p = 0.02). The prevalence of aborted SCD before ICD implantation was not significantly different in both groups, three patients with ICD therapy versus one patient without ICD therapy. Time from diagnosis to implantation was of no significance.

**Table 2:** Demographic and clinical parameters of both patient groups.

Clinical characteristics	ICD therapy	No ICD	Odds ration	p-value
	n = 12	therapy n = 14	(95% CI)	
Men	11	10	4.4 (0.42, 46.3)	0.217
Family history of ARVC	1	3	0.33 (0.03, 3.72)	0.372
Age at diagnosis (years)	39.5	39.5	1.00 (0.94, 1.06)	0.968
	(35.0, 54.0)	(36.8, 48.5)		
Follow-up duration (years)	14±10	9.8±9.7	1.01 (0.99, 1.02)	0.202
Symptoms at time of				
ARVC diagnosis				
- aborted SCD	3	1	4.0 (0.36, 45.1)	0.262
- syncope	3	9	0.11 (0.018, 0.7)	0.020
- heart failure	1	0	0.28	0.48
			(0.01, 7.67)fis*	
- VT	10	9	2.22 (0.33, 15.18)	0.415
History of heart failure	4	0	15.35	0.033*
			(0.73, 321.60)*	
Arrhythmia				
- AFib or flutter	2	1	0.60 (0.047, 7.63)	0.694
- VT	8	7	0.667 (0.13, 3.35)	0.622
- Other (WPW, AVNRT)	2	1	0.60 (0.047, 7.63)	0.694
Drug therapy				
- betablocker	9	5	0.397 (0.08, 1.94)	0.253
- Amiodarone	3	2	0.227 (0.022, 2.4)	0.217
- ACEI or ARB	4	1	0.227 (0.022, 2.4)	0.217
ICD				
- primary prevention (vs	0	2	2.78 (0.1, 74.7)	0.99
secondary)				
- time from ARVC	3.25	0.06	0.90 (0.76, 1.06)	0.19 (0.067 M–W)
diagnosis to ICD	(0.06, 21.5)	(0.004, 1.23)		
implantation (years)				
- age at time of	52	39	1.075	0.063(0.053 M–W)
implantation	(39.3, 60.5)	(34.8, 50.5)	(0.996, 1.16)	

* p-values based on Fishers exact testing.

Summaries within groups are given as number with the feature for categoric variables and median (inter-quartile range) for numeric data.

(**VT**: ventricular tachycardia; **WPW**: Wolff-Parkinson-White-Syndrome; **AVNRT**: atrioventricular nodal re-entrant tachycardia; **ACEI**: angiotensin-converting enzyme inhibitor; **ARB**: angiotensin receptor blocker).

Four patients (33%) having received ICD therapy had a previous history of heart failure, whereas none of the patients without ICD therapy during follow-up had a history of heart failure. Using Fishers exact test this difference is significant (p = 0.033) and using exact methods to create a confidence interval for the difference (4.3%, 60.9%) also excludes compatibility with zero difference. The time period elapsed between ARVC diagnosis was made and ICD implantation was different for both groups: In patients who received an appropriate ICD therapy during follow-up the ICD was implanted a median time of 3.25 years after diagnosis, while patients who did not require an ICD therapy which had the ICD inserted after a median of 3 weeks (p = 0.19; n.s.). During follow-up two patients died (due to infective endocarditis and end-stage heart failure after heart transplantation). One patient required an external biventricular assist device because of severe heart failure and one patient was lost to follow-up.

### Transthoracic echocardiography

The transthoracic echocardiography revealed left ventricular involvement in 12 patients: 8 (67%) patients out of the patient group with appropriate ICD therapy compared to 4 (29%) in the group without appropriate ICD therapy (p = 0.059). The right ventricle most commonly showed the typical sites of disease predilection, which are the right ventricular apex, the inflow and outflow tracts, the so-called “triangle of dysplasia” [7]. All patients with appropriate ICD therapy presented the typical RV aneurysm compared to 11 (85%) in the other group. The detailed echocardiographic findings are listed in table 3.

**Table 3:** Echocardiographic, electrophysiologic study and ECG parameters.

Echocardiographic	ICD therapy	No ICD	Odds ration	p-value
parameters	n = 12	therapy n = 14	(95% CI)	
LV involvement	8/12	4/14	5.00	0.059
			(0.942, 26.53)	
LA dilatation	3/11	3/11	1.00	0.99
			(0.153, 6.531)	
RA dilatation	6/11	7/12	0.857	0.855
			(0.164, 4.467)	
RV aneurysm	12/12	11/13	0.307	0.6513
			(0.0054, 4.624)	
RV dilatation	7/11	4/13	3.937	0.114
			(0.718, 21.594)	
RV systolic function	1/12	5/13	0.16	0.125
impairment			(0.015, 1.66)	
RV area (cm^2^)	29.5 (20.8,	22.0	1.104	0.189
	36.5) n = 10	(17.5, 27.0)n = 5	(0.952, 1.279)	(0.193 M–W)
RV fac (%)	32 (25, 46)	25	1.067	0.173
	n = 9	(23, 35.5) n = 8	(0.972, 1.172)	(0.173 M–W)
RV TAPSE (mm)	18 (16.25, 22)	17 (10.25,	1.094	0.326
	n = 8	21.5) n = 8	(0.914, 1.309)	(0.544 M–W)
**Electrophysiologic study and ECG parameters**				
EP study performed	9/12	7/14	3.0 (0.56,16.01)	0.20
QRS >120 msec	7/10	4/12	4.67 (0.77,28.5)	0.10
PR >200 msec	3/10	2/13	2.36 (0.31,17.8)	0.41

(**LV**: left ventricle; **LA**: left atrium; **RA**: right atrium; **RV**: right ventricle; **fac**: fractional area change; **TAPSE**: tricuspid annular motion).

Summaries within groups are given as number with the feature for categorical variables and median (inter-quartile range) for numeric data.

### ECG parameters

No significant differences in both groups were observed with respect to PR prolongation (>200 ms) or QRS duration (>120 ms).

### Invasive electrophysiological study

The procedure was performed in 9 patients of patients with appropriate ICD therapy, and 7 patients without ICD therapy. Sustained ventricular tachycardia could be induced in 8 (89%) patients of the first group, and in 7 (100%) patients without shocks. Catheter ablation for a sustained monomorphic ventricular tachycardia was performed in one patient of each group.

### Complications and mortality

No early (<30 days after ICD implantation) complications occurred in our patient population. Two late complications related to the lead were observed: A 51-year-old physically active man who performed Nordic walking on a regular basis and never required an ICD therapy developed electrode dysfunction with signal undersensing due to insulation failure 12 years after the implantation. The second patient was a 61-year-old man who presented with bacterial endocarditis with vegetations on the ICD lead one year after implantation requiring explantation of the lead and the generator. Subsequently, this patient died as a consequence of acute low output failure due to electrical storm which was refractory to drug therapy and external shocks. Another patient died due to progressive congestive heart failure three years after heart transplantation without replacing the ICD.

### Multivariable analysis

Among all clinical variables analyzed, the presence of the history of heart failure was identified as a predictor of a subsequent appropriate ICD therapy. LV involvement and the age at implantation were borderline significant predictors in our patient cohort. The presence of syncope at the time of diagnosis was more likely to be found in patients who were not shocked (p = 0.022). Invasive electrophysiological testing was not significantly associated with appropriate ICD therapy.

## Discussion

The clinical course of ARVC varies considerably. Cardiac electrical instability may lead to life-threatening ventricular arrhythmias and SCD. Therefore, ARVC patients are often considered for ICD implantation after a survived SCD or after a presentation with syncope related to ventricular tachyarrhythmias [13]. However, little data are available to guide the prophylactic indications for ICD implantation in ARVC patients.

During a median follow-up duration of 10.7 years, 12 patients (46%) with ARVC received appropriate ICD therapy, 9 of whom within the first two years after device implantation. Other studies with a considerably shorter observation period documented similar rates of appropriate ICD therapy. Corrado et al showed appropriate ICD therapies in 64 patients out of 132 patients (48%) during a follow-up period of 3.3±2.3 years [29]. Wichert et al reported appropriate ICD therapies in 70% of 60 patients observed over an interval of 6.7±3.6 years [30].

In the present single-center patient cohort, history of heart failure was a significant predictor of appropriate ICD therapy during long-term follow-up. Left ventricular disease involvement and time between ARVC diagnosis and ICD implantation were of borderline significance. Invasive electrophysiological testing did not predict appropriate ICD therapy. The findings of this study are consistent with the results of the previously published report from our group, in which we were able to demonstrate that congestive heart failure and left ventricular involvement were independently associated with adverse outcome in 61 patients with ARVC, including 24 patients who were implanted with an ICD [32]. Komura et al reported that the onset of heart failure was associated with significantly higher mortality than the onset of ventricular tachycardia in 35 ARVC patients [33]. Left ventricular involvement is not a major diagnostic characteristic for ARVC; it may rather be a variant of ARVC or a sign of disease progression, and hence, as reported here, an important predictor of arrhythmic ventricular events and ICD therapy. However, ARVC is characterized by electrical instability of the right ventricle presenting ventricular arrhythmias of right ventricular origin. Therefore, the role of an arrhythmogenic co-factor of left ventricular involvement of the disease remains to be established, but may just reflect a greater involvement of total myocardial mass by the disease process.

Besides a left ventricular involvement, the echocardiographic evaluation revealed no major differences between patients with or without appropriate ICD therapy. Indeed, right ventricular dimension and function were similar between the two groups. However, two-dimensional echocardiography for right ventricular evaluation is limited by the complexity of the right ventricular anatomy not resembling simple geometrical shapes and this may have confounded the study result. In addition, since left ventricular involvement is often associated with regional echocardiographic anomalies, the assessment of the ejection fraction using the biplane area-length method may have failed to demonstrate such abnormalities [28].

Previous studies investigating the predictive value of syncope for appropriate ICD therapy have yielded conflicting results [34], [35]. In the present study, we could not confirm any predictive value of syncope as reported by some studies. We might have been unable to confirm this due to the small sample size of our cohort. Our study examined 26 patients, whereas Komura et al studied 35 patients and Corrado et al 106 consecutive patients.

Another explanation may be that syncope may not be of arrhythmic origin in some patients and linked to hemodynamic factors.

In the majority of our patients ventricular tachycardia or fibrillation could be induced during electrophysiological stimulation. However, arrhythmia inducibility was not significantly associated with appropriate ICD therapy, although the confidence intervals were wide and we cannot exclude odds ratios of over three for VT and over seven for the other two components due to the small patient number. Nevertheless, these findings are in accordance with those of Piccini et al. [19] who enrolled 67 patients and found that electrophysiologic testing did not predict appropriate ICD interventions in patients who received an ICD for primary prevention.

### Study Limitations

This is an observational study, and hence, it is not possible to ascribe causality. Although the study cohort was relatively large, given the rarity of ARVC in the population at large, and the follow-up period important for an ARVC population of a single-center, the major weaknesses of this report are those inherent to observational trials in general. Another limitation might have been the concomitant use of antiarrhythmic medication that can impact on the rate of appropriate ICD therapy [31]. In this cohort, betablockers were used more commonly than sotalol or amiodarone considering the young age of the patient group. However, there was no significant difference in antiarrhythmic medication at baseline and no significant change during follow-up between the two groups.

### Conclusions

History of heart failure is a significant predictor of appropriate ICD therapy in patients with ARVC. Therefore, close clinical monitoring of these patients is important and signs of heart failure should alert the treating physician. More long-term studies on clinical variables influencing the outcome of patients with ARVC in larger multicenter cohorts currently developed are warranted in the future.
